# Impacts of a Clinical Research Program for High School Students from Groups Historically Excluded from Science and Medicine

**DOI:** 10.1089/heq.2022.0050

**Published:** 2022-11-25

**Authors:** Laura K. Krecko, Sarah Jung

**Affiliations:** Department of Surgery, University of Wisconsin School of Medicine and Public Health, Madison, Wisconsin, USA.

**Keywords:** STEMM, underrepresented, diversity in medicine, pathway program

## Abstract

**Introduction:**

Pathway programs engage students who identify with groups historically excluded from, and therefore underrepresented in, science, technology, engineering, math, and medicine (STEMM). We explored alumni-reported impacts of eight U.S. high school-to-college pathway programs funded by the Doris Duke Charitable Foundation (DDCF).

**Methods:**

A survey was sent to 499 alumni to evaluate their perceptions of the DDCF programs, which offer mentored experiences in clinical research. A multivariate analysis was used to compare Likert-style questions related to the programs' impact on education and career plans. Open-ended responses were analyzed using inductive analysis.

**Results:**

Two hundred sixty-nine alumni responded to the survey, the majority of whom identified as Hispanic/Latinx or Black/African American. One hundred nineteen alumni (∼75%) currently in college reported majoring in STEMM fields. Of college graduates, 30 (∼65%) obtained a degree in an STEMM field. Participants identifying as Hispanic/Latinx had a significantly higher reported level of impact of the programs on their plans to attend college. Most alumni felt that the programs impacted their chosen majors and future professions and made them more confident to pursue careers in STEMM.

**Discussion:**

Surveyed alumni perceive DDCF programs to have positively impacted their interest, confidence, and skills in STEMM-related areas. Our results support the benefit of DDCF programs and substantiate their funding, integration into higher education systems, and iterative redesign to ensure positive impacts on students with diverse backgrounds.

**Health Equity Implications:**

Assessment and improvement of pathway programs may support underrepresented students in their STEMM aspirations and increase the diversity of the medical and scientific workforce.

## Introduction

Diversity in the medical workforce improves the care provided to a wide range of patients.^1,2^ To promote diversity within the medical field, it is critical to recruit and retain students, trainees, and providers from a variety of backgrounds.^3^ Unfortunately, there remains a discrepancy between the demographic makeup of the U.S. population and the backgrounds of individuals accepted to medical school and currently in medical practice.^4^

The demographics of actively practicing physicians are not representative of the U.S. population as a whole, with proportionately fewer practicing physicians who identify as Black/African American, Hispanic/Latinx, and American Indian/Alaska Native.^5,6^ These statistics reflect the impact of structural racism—including bias and discrimination in medical education and recruitment—on health care and emphasize the need to combat systemic inequity by taking deliberate and early actions to recruit individuals from historically marginalized groups into the medical workforce.^7,8^

Pathway programs have been developed to increase the number of underrepresented students in the training pathway of fields such as medicine and surgery.^9^ Pathway programs are designed to recruit and retain students who identify with groups historically excluded from, and therefore underrepresented in, science, technology, engineering, math, and medicine (STEMM) fields, including Black/African American, Hispanic/Latinx, and American Indian/Alaska Native students, students with lower socioeconomic status (SES), female students, and first-generation college students.^10,11^

Pathway programs emphasize early exposure to science and medicine, research and clinical skill acquisition, mentoring relationships, peer community building, professional development, and development of academic skills.^12–15^ Such programs have been shown to positively impact students' intentions to pursue science and medicine, as well as their confidence and self-efficacy in entering these fields.^16,17^ Objectively, these programs have been found to increase pursuit of undergraduate majors, graduate training programs, and subsequent careers in STEMM for students with diverse backgrounds.^18,19^

Although these programs may improve important predictors of academic success such as self-efficacy and feelings of belonging in academia,^20^ even with these interventions, students from underrepresented groups are less likely to complete medical and science-focused degrees compared with their nonunderrepresented peers.^11,21,22^ Further work is therefore warranted to better understand how to design these programs to more fully support students from underrepresented groups in their desired STEMM trajectories.

Although some barriers faced by underrepresented students cannot be mitigated by pathway programs alone, such as structural factors perpetuating systemic bias and discrimination, given the known beneficial outcomes associated with pathway program participation, it is important to evaluate programmatic aspects that can be targeted for improvement. As such, there is a need to closely assess the value of these programs and their potentially differential impacts on students with various backgrounds.

This article explores alumni-reported impacts of eight U.S. pathway programs funded by the Doris Duke Charitable Foundation (DDCF), which enroll students from underrepresented groups who have an interest in STEMM. The question driving this work was “How do program alumni perceive their experiences in these programs and the impact of these programs on their college and career choices?”

## Methods

This study was approved by an Institutional Review Board. Survey data were collected from alumni of eight high school-to-college pathway programs throughout the United States, all of which received funding from the DDCF (see https://www.ddcf.org/funding-areas/medical-research/clinical-research-continuum-high-school-to-college/ for a list of program sites). The programs were located in cities in the west coast, Midwest, and east coast areas.

Eligible students included high school students from populations meeting the National Institutes of Health's definition of underrepresented in the extramural scientific workforce.^22^ Most programs were conducted as summer research programs between students' junior and senior years of high school. All programs provided students with a mentored experience in clinical research.

A survey that assessed alumni perceptions of the programs and current education and career trajectories was sent to 499 alumni who completed the programs between 2012 and 2019 and for whom the research team was able to verify contact information. Open-ended questions were crafted using a formative evaluation perspective to gather data on what makes the program successful and should be continued, as well as on potential mechanisms to improve program operations and procedures.^23^

Survey responses were collected between February 25, 2020, and April 1, 2020. A multivariate analysis was used to compare six survey questions related to the programs' impacts on plans to attend college, chosen major, and future profession and confidence to pursue careers in STEMM. Questions were constructed using Likert scales ranging from 1 (not at all) to 5 (a great deal) or from 1 (much less confident) to 7 (much more confident). Qualitative themes from open-answer comments were summarized to enrich the quantitative survey results.

Open-ended responses were analyzed using an inductive analysis, whereby one researcher with a background in sociology and education analyzed the responses to each of the open-ended questions and categorized them into themes. These themes were reviewed with a second member of the research team with a background in psychology and education.

An important component of the qualitative analysis is the reflexivity of the researchers or our positionality as researchers with backgrounds in sociology, psychology, and education, as well as our personal experiences, such as that of a first-generation college student in the case of the educational psychologist. This is important to acknowledge given that while the goal of the data collection and analysis was to represent the experiences and perspectives of the alumni, our positionality undoubtedly influenced the questions that were asked and the themes that were developed from the data.

## Results

### Demographics

Of the 499 alumni invited to take our survey, 269 responded (response rate 54%). The mode age of survey respondents was 19 years and the mean was 20.9 (range 18–26) years. The majority of alumni self-identified as Hispanic/Latinx or Black/African American (Table 1). Reported levels of parental/guardian education were variable and summarized as follows (with ranges presented given that alumni were asked to report for up to two parents/guardians): less than a high school level of education (20–24%), high school diploma or GED (15–18%), bachelor's degree (16–20%), and graduate/professional degree (17–21%).

**Table 1.** Alumni-Reported Race and Ethnicity

Race and/or ethnicity	Number reporting
American Indian or Alaska Native	2
Asian	41
Black or African American	73
Hispanic or Latinx	105
Native Hawaiian or Other Pacific Islander	8
White	16
Middle Eastern	6
Black/White	7
Black/Asian	2
Asian/White	2
Chose not to report	7

There was variability in household income, with the mode reported household income between $25,001 and $50,000 (59 respondents, 21.9%). One hundred sixty-nine respondents (62.8%) reported an annual household income of $100,000 or less; however, 56 respondents (20.8%) reported an average household income of over $100,000.

### Undergraduate education and STEMM persistence of alumni

Overall, alumni felt that the programs impacted their chosen college majors and future professions and that the programs made them more confident to pursue careers in clinical research and STEMM (Table 2). According to the analysis of covariance results, when asked about the impact of the programs on the decision to go to college, even after controlling for parental education and household income, there was a significant difference in the reported level of impact of the program on plans to attend college based on race/ethnicity, *F*(3, 188)=5.257, *p*=0.002, and *η*^2^*p*=0.077, a medium effect.

**Table 2.** Descriptive Statistics for Questions Related to Future Major and Profession and Confidence to Pursue Careers in Clinical Research, Medicine, and Science, Technology, Engineering, Math, and Medicine

Question	***N***	Mean	Standard deviation
Impact of the program on your chosen major	247	3.52	1.259
Impact of the program on your future profession	246	3.67	1.219
Impact your confidence to pursue a career in clinical research	265	5.57	1.548
Impact your confidence to pursue a career in medicine	265	5.42	1.654
Impact your confidence to pursue a career in an STEM field	265	5.78	1.500

**FIG. 1.**
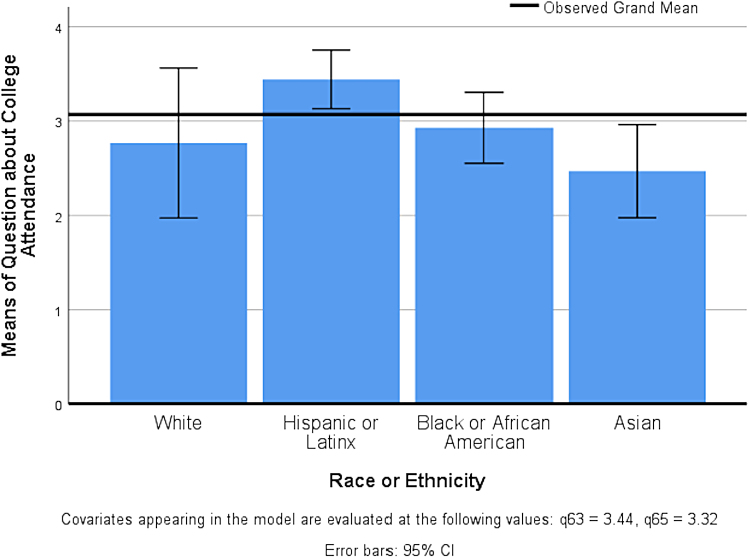
Differences in reported impacts on plans to attend college by self-identified race or ethnicity. Likert scale scoring: 1 = not at all, 2 = a little, 3 = somewhat, 4 = quite a bit, and 5 = a great deal. CI, confidence interval.

Follow-up tests, using the Bonferroni adjustment for multiple comparisons, showed a significant difference between Hispanic/Latinx participants and Asian participants in the programs' impact on their plans to attend college, *p*<0.05 and *d*=0.65, a medium effect (Table 3). See Figure 1 for a representation of differences between the groups and comparison with the grand mean. There were no meaningful differences in alumni-reported impacts on chosen major, future profession, and confidence to pursue careers in STEMM based on parental education, household income, race or ethnicity, or program (Table 2).

**Table 3.** Descriptive Statistics by Reported Race or Ethnicity for the Question on How Much the Program Impacted the Decision to Go to College

Reported race or ethnicity^a^	***N***	Mean	Standard deviation
White	15	2.47	1.685
Hispanic or Latinx	98	3.52	1.452
Black or African American	65	2.85	1.660
Asian	38	2.53	1.606

^a^ Some categories of race or ethnicity did not have a large enough sample to include in the model.

Of the 269 respondents, 160 (∼60%) were currently enrolled in a 4-year college or university. Overall, 119 alumni (∼75%) currently in college or university reported majoring in STEMM fields. Forty-six alumni (∼20%) reported having graduated from college or university. Of these graduates, 30 (∼65%) reported graduating with a degree in an STEMM field.

Alumni currently in college majoring in an STEMM field or who graduated with a major in the STEMM field described five major themes related to their motivations for pursuing STEMM: (1) enjoying STEMM in general and wanting to contribute to science; (2) wanting to work in a medical or health care field; (3) being interested in a specific research field in STEMM (e.g., biology or chemistry); (4) developing an interest in STEMM and the process of discovery through their research experience; and (5) experiences in the programs reinforcing that STEMM fields need more diversity.

Alumni were asked to describe which parts of the programs impacted them the most and to explain how they were impacted. The following 10 categories summarize areas of greatest reported impact: (1) learning about research and the research process; (2) opportunities to engage in hands-on learning; (3) mentorship from researchers, clinical practitioners, and others (such as graduate and medical students); (4) communication skill development, particularly the opportunity to read, write, and present scientific findings; (5) confidence in their skills and abilities to pursue their careers of interest; (6) having a unique opportunity that offers advantages and challenges; (7) experience working in a laboratory; (8) career exploration; (9) data collection and analysis experience; and (10) clinical experiences.

### Continued engagement with the programs and recommendation to others

The majority of alumni respondents were interested in opportunities to continue to engage with the programs as alumni. Alumni indicated interest in continuing to engage in the programs in multiple ways, including (1) attending networking or reunion events; (2) participating in panels to reflect on their program experiences and provide updates about their career trajectories; and (3) participating in research projects if there were further opportunities through the programs.

Alumni independently suggested a variety of other ways they could potentially engage with the program following program completion, including engaging other alumni and current participants through social media, providing administrative support or help with website design, and making monetary donations.

Moreover, alumni suggested that they could mentor students in some of the skills they have learned, particularly in regard to navigating the college/university process, through in-person or virtual mentoring. One hundred forty-two alumni reported they would be “very” or “extremely” interested in mentoring new students in the programs.

Resources that respondents thought could be of most help if added as part of the programs were an online forum to engage with other program alumni (given that 176 alumni reported remaining in informal contact with other members of their cohorts); more ways to reach back out and contact the programs following program completion; and access to counseling for school or personal issues that make degree completion difficult.

When asked if they would recommend the programs to other students, alumni responses were overwhelmingly positive and reflected six distinct themes supporting reasons for program recommendation: (1) research experience; (2) developing skills in areas of research, communication, and leadership; (3) exploration of different careers and majors; (4) opportunity to network and connect with those working in the medical field, which they might not otherwise get; (5) developing confidence in themselves; and (6) programmatic elements, including mentorship, relationships with other students, and the positive program environment.

## Discussion

We surveyed alumni of eight high school-to-college clinical research pathway programs funded by the DDCF throughout the United States to assess their current career trajectories and explore their perceptions of the programs. Our results suggest positive impacts of the programs on STEMM persistence, with 75% of responding alumni who are currently in college reporting majoring in an STEMM field and 65% of those who have graduated from college reporting having received an STEMM-related degree.

Our results also indicate that program alumni perceive the programs to have positively impacted their interest, confidence, knowledge, and skills in STEMM-related areas and to have reinforced their appreciation for the importance of increasing diversity in STEMM. Moreover, many alumni indicated interest in continued engagement with past and present program participants. The ability to recognize and respond to the experiences and identities that students bring with them into the academic setting is an important factor in mentoring relationships.

As such, engaging interested alumni as mentors may prove helpful for program participants who are seeking mentorship. It will be important, however, to ensure that mentors are appropriately compensated for their time to avoid contributing to the “minority tax” phenomenon, in which individuals from underrepresented groups are asked to assist with recruitment and other diversity, equity, and inclusion efforts without adequate recognition.^24^

Systemic racism and discrimination have resulted in inequitable pursuit of and access to careers in the medical and scientific workforce for individuals from groups historically excluded from STEMM fields. Pathway programs have been increasingly developed and implemented to increase matriculation of students from historically underrepresented groups into these fields.

While our survey results are promising and suggest an overall positive impact of the evaluated programs, pathway programs have limitations that represent areas in need of improvement.

First, programs may differentially impact participants from different groups and backgrounds. We found that participants identifying as Hispanic/Latinx had a significantly higher reported level of impact of the DDCF programs on their plans to attend college. More research is needed to understand this differential impact. Previously characterized educational barriers faced by some Latinx youth include low SES, low level of parental education, financial challenges, lack of knowledge about potential careers, language differences, discrimination related to immigration/legal status, lack of access to resources, and exposure to negative attitudes in academic settings.^25–27^

Given these challenges, researchers evaluating education for Latinx students have highlighted the importance of engaging parents, families, and communities in educational efforts,^25,26,28^ promoting early exposure to STEMM topics using hands-on approaches,^28^ and ensuring that students perceive equitable treatment.^27^

DDCF programs actively encourage perceptions of equitable treatment through the emphasis on all students' abilities to succeed in science and medicine, and a positive program environment was one of six themes we identified in the present study for why respondents would recommend the programs to others. Furthermore, the DDCF programs promote hands-on exposure to STEMM fields: this was one of the 10 major areas of programmatic impact reported by alumni in our survey.

Future program iterations, however, should consider additional factors such as community and family engagement and other culturally sensitive strategies to continue to address specific challenges faced by participants. This is particularly important for Black, female, and low-SES students who, like Hispanic/Latinx students, are less likely to maintain interests in STEMM careers throughout high school.^11^

Examples of programs that have positively impacted Black and female students include USSTRIDE (Undergraduate Science Students Together Reaching Instructional Diversity and Excellence), an undergraduate program with increased associated acceptance rates of Black and Latinx students to medical school,^29^ and the Health Disparities Summer Internship program, which resulted in more than half of the 73 participating students (69.9% of whom were Black and 68.5% of whom were female) reporting an increase in confidence to pursue a research or medical career.^30^

It will be critical to evaluate aspects of the DDCF programs that can be modified to ensure a positive impact on other groups of students who did not report as great an impact of the program on their career aspirations.

Of note, while we and others strive to identify common barriers faced by students from underrepresented groups with aspirations in STEMM, the same challenges cannot be generalized to all students^27^ and there is no single model that can address all the diverse needs of students.^31^ This reinforces the importance of programs assessing individual participants' needs, goals, and aspirations, acknowledging the range and variety of experiences that each student brings.

Pathway program research also emphasizes the importance of maintaining culturally inclusive practices to address cultural and societal barriers to STEMM participation and to improve the educational environment for students from underrepresented groups.^31,32^ Ensuring similarly culturally sensitive programming may be an important next step for the DDCF programs.

A second potential area for continued focus in our pathway programs is participant and mentor recruitment. Students at the intersection of female, Black, Hispanic, and low-SES identities have lower rates of maintaining aspirations in STEMM throughout high school,^11^ yet some pathway programs do not have adequate minority representation.^30^ Students identifying with these groups may be most likely to benefit from program participation and mentorship and therefore these individuals, particularly those representing dual or triple underrepresented statuses,^11^ should be deliberately recruited.

Third, structural alignments with other pathway programs may help increase their relative impact. Wilbur et al^2^ recommend establishing partnerships between colleges, universities, and medical schools to strengthen pathway programs for underrepresented students. Based on this recommendation, additional work in establishing and strengthening existing relationships with undergraduate and medical schools to ensure continuity for students throughout their training and career paths is warranted.

Furthermore, despite long-standing evidence for the effectiveness of pathway programs, programs face waning governmental and philanthropic financial investment.^33^ Our results corroborate the positive impact of these programs and substantiate the need for their continued funding and integration into systems of higher education.

Finally, it is essential to acknowledge that a key limitation to these programs is that they do not mitigate all external factors that influence participants' future career trajectories, such as the bias and discrimination in recruitment for scientific and medical training^8^ and other aspects of structural racism. For this reason, it is clear that pathway programs represent one of many larger societal changes necessary to enact change.

Additionally, while our results about the impacts of the programs are overwhelmingly positive, 3 of the 269 alumni respondents said they would not recommend the program to other students. The open-ended responses of two of these alumni revealed that this was due to issues of not feeling connected with and supported by program staff. Furthermore, we do not have information about the demographics and experiences of the alumni who chose not to respond to the survey.

### Health equity implications

Continuing to assess and improve pathway programs is an essential component of supporting students from groups historically excluded from and therefore underrepresented in STEMM. To best serve our diverse communities, educational systems must facilitate equitable access to training in STEMM for individuals from historically underrepresented groups, promoting not only their application and matriculation into STEMM programs but also their retention and promotion.

The continued study, improvement, and funding of pathway programs will serve as one strategy—of many that are necessary–to ensure the continued support of students from persistently underrepresented groups in their STEMM career aspirations.

## Conclusions

Alumni of eight DDCF high school-to-college clinical research pathway programs reported positive perceptions and impacts of program participation, including increased interest, knowledge, confidence, and skills, as well as increased intentions to pursue a career in STEMM. Alumni identifying as Hispanic/Latinx particularly felt the program impacted their intent to matriculate to college, and the majority of alumni wished to engage in future program iterations through mentorship.

These findings support the beneficial role of pathway programs in increasing diversity and equity in health care, which is essential for provider well-being and quality of patient care. Pathway programs require continued study, redesign, and funding to remain impactful and beneficial for all participants.

Furthermore, while these programs appeared to have an overall positive impact on participants, they represent only one of many levels of interventions necessary to dismantle systemic racism to improve equity and diversity in STEMM.

## Abbreviations Used

CI: confidence interval
DDCF: Doris Duke Charitable Foundation
SES: socioeconomic status
STEMM: science, technology, engineering, math, and medicine
